# Supplementary material for: Anemia or other comorbidities? using machine learning to reveal deeper insights into the drivers of acute coronary syndromes in hospital admitted patients
Source: PLoS One. 2022 Jan 24;17(1):e0262997. doi: 10.1371/journal.pone.0262997 (PMC8786175; doi:10.1371/journal.pone.0262997)
Supplement: Attachment — Submitted filename: Response_to_Reviewers.docx [file pone.0262997.s001.docx]

Reviewer #1 (note that revisions starts after the new figures below):

*1- However, the authors are advised to completely rethink the presented analysis.
By their machine learning approach the authors found that length of hospitalization and haemoglobin at admission were the most important features for predicting the risk of mortality, heart failure, and bleeding. They further report that “risk of all ACS outcomes increases when patients stay in the hospital for more than 10 days“. This is one of the major limitations of the study: Length of hospital stay is an outcome parameter rather than a predictor and should not be used as a predicting parameter for events during the same hospitalization (the authors count their outcome parameters death, heart failure, bleeding only as long as the patient is still in hospital). In case of death, this event ends hospitalization and thus has a direct influence on its predictive parameter length of stay. Similarly, patients experience their in-hospital heart failure and in-hospital bleeding before the value of their predicting variable “length of hospital stay” can be known. In addition, in-hospital heart failure and bleeding have a direct influence on length of stay: In most patients they would prolong hospitalization so that length of stay is a function of heart failure and bleeding and not vice versa. This is in my opinion misconception that also invalidates further calculations which are based on length of stay being among the three strongest predictors. Therefore, I do believe that this manuscript cannot be accepted for publication in the Journal.*

- We agree with the reviewer’s point in terms of excluding length of stay from the analysis. Hence, we have repeated the whole analysis and revised all the results and figures. While removing length of stay slightly dropped the performance of all selected machine learning algorithms, the initial hemoglobin levels remained as the strongest predictor and interacting variable with other predictors. Therefore, we hope that the reviewer now sees the revised version acceptable for publication and thank them for their valuable time in reviewing the manuscript.

*2- The authors need to elaborate more on the rationale of the research question and the use of machine learning for their specific research question.*

- We elaborated extensively in both introduction (lines 45-74, 101-106 & 107-116) and discussion sections (lines 326-329, & 347-359) for the rational of using ML for our specific research question as suggested by the reviewer.

*3- The authors should put more emphasis in explaining the clinical impact of their results and the use of machine learning tools.*

- We extensively revisited the explanation of the clinical impact of our results derived from ML Model as suggested by the reviewer.

*4- The authors should revise the manuscript – including language - to improve readability. The manuscript text is interrupted by a table an various figure legends, which hampers understanding such a complex analysis. Furthermore, I did not have access to supplemetary table 1.*

- We extensively revised the linguistics of the manuscript as suggested by the reviewer. However, the presence of tables and figure legends in the body of the manuscript is due to following the journal’s strict submission guidelines. We also apologize for miss-referencing supplementary table 1 as it was referring to table 1.

*5- Figure 2: The authors state that the risk for death, heart failure, and bleeding markedly increases after 10 days of hospital stay. I can confirm this statement for heart failure and bleeding. However, for mortality it looks like a steady rather than an abrupt increase.*

- The point is no longer valid, after removing length of stay from the analysis as suggested by the reviewer.

*6- The authors state that the risk of heart failure increases at the age of 50 years and above. However, in figure 2H it looks as if the risk of heart failure does not increase below 60 years and markedly increase around 70 years and older.*

- After removing length of stay from the analysis the results slightly changed, and we commented extensively on this point as suggested by the reviewer (lines 299-305).

*7- The Authors state that patients with Hb levels <10g/dl are at a higher risk of death, heart failure, or bleeding. The risk of heart failure, however, increases already at higher haemoglobin levels ( <13g/dl?). See also Figures 3 F and 3 I. The authors should comment on these discrepant findings.*

- We extensively commented on this as suggested by the reviewer (Lines 313-322).

*8- I believe figure 2 would benefit from using the same scale on all y-axes for better comparison.*

- We attempted to unify the y-axes for all figures as suggested by the reviewer. However, some of the resulting figures looked distorted with large empty spaces, as the probability thresholds differs between important features. Yet, the x-axis is unified among the same features.

Reviewer #2 (note that revisions starts after the new figures below):

*1- One major concern is that the study seems to lack a specific hypothesis and scientific question and therefor is more a report of multiple incoherent findings rather than a comprehensive paper. In the abstract and the conclusion the authors heavily emphasize their findings on anemia and ACS and claim that they “revealed the unique and complex role of anemia on admission in the prognosis of different ACS outcomes”. However the majority of the presented data are on the role of length of hospital stay and age.*

- We made substantial revisions throughout the manuscript in this regard. We made the paper more oriented toward how machine learning is more effective than classical risk stratification tools used by most clinicians in revealing complex relationships between different risk factors and ACS in-hospital events. Then how our selected ML models can produce rich outputs that is more interpretable than past used ML models. Finally, we showed how our ML untangled the complex relationship between initial hemoglobin values at admission and different common in-hospital ACS events with an extensive and elaborated discussion. We hope that the current version is more acceptable to the reviewer and we thank them for their time in reviewing the manuscript and provide useful suggestions.

*2- Furthermore the findings on anemia are not reported or discussed in sufficient depth. In regard to Figure 2 the authors state “[…] patients with initial hemoglobin values of equal or less than 10 g/dl are more likely to be discharge dead, had heart failure or develop bleeding”. While this seems to be true for mortality (Fig 2 D), the curve in Figure 2 E for heart failure seems to be u-shaped with a high risk at an Hb around 10 but lower risk at Hb 5-9 and 11-15, which is not addressed in the manuscript.*

- We extensively commented on this as suggested by the reviewer (lines 313-322).

*3-* *Another major concern is with the underlying data which were analyzed. In table 1 the number of Patients receiving PCI is reported which is 0.35 % of the 4044 Patients with ACS. This rate of PCIs in an ACS collective seems surprisingly low. Also the type of ACS is not further evaluated in the manuscript. I think it would enhance the quality of the manuscript to evaluate the impact of the different hemoglobin levels in the different types of ACS. In addition it would be important to get information on how many values actually were present in regard to the single characteristics and outcomes.*

- We did comment on this in the discussion section as suggested by the reviewer (lines 331-340). Further, the evaluation of other types of ACS will make the content of the manuscript complicated and distracting to the reader. Yet, we commented on this in the discussion section. Thus, we think that current version of the manuscript is already rich and extensive.

*4- Line 142: “Heart failure was the third most important feature for predicting ACS mortalities […]” Does heart failure in this context mean heart failure as a preexisting condition or a complication?*

- It is an in-hospital heart failure which different from prior CVD. We clarified this and made it distinct throughout the manuscript.

5- Line 205-207: The authors discuss that length of stay is probably dependent on complications instead of being an independent factor toward predicting the risks of ACS. I agree with the authors but I think that raises the question why length of stay is addressed as a risk factor and not as an outcome. I think it would make more sense to analyze it in that way.

- We removed length of stay from the models as suggested by reviewer 1 and repeated the whole analysis. Yet, we agree with the reviewer’s notion of setting length of stay as an outcome. However, the manuscript is already rich in content after the substantial revision. Thus, building a model for length of stay might complicate the results and distract the reviewer. Yet, we commented on this in the discussion section (Lines 367-372).

6- Figure I In Figure I the Abbreviation CRF is used without being explained in the manuscript

- Abbreviation CRF has been spelled out in all of the figures as suggested by the reviewer.

7- Figure 3 B, E, H; Please explain more detailed what those graphs are actually showing.

- We described and interpreted these figures as suggested by the reviewer.
