# Supplementary material for: Anemia or other comorbidities? using machine learning to reveal deeper insights into the drivers of acute coronary syndromes in hospital admitted patients
Source: PLoS One. 2022 Jan 24;17(1):e0262997. doi: 10.1371/journal.pone.0262997 (PMC8786175; doi:10.1371/journal.pone.0262997)
Supplement: Attachment — Submitted filename: Response_to_Reviewers.docx [file pone.0262997.s002.docx]

Reviewer #2:

*In the revised manuscript, the authors made substantial changes to both the analysis (by excluding length of stay and rerunning the analysis) and the writing of the text. Overall the applied changes do significantly enhance the quality of the manuscript. There are only some minor comments which I would like to make:*

- We thank the reviewer for their valuable time and comments, which substantially improved the quality of the manuscript and hope that the minor requested revisions were fulfilled as suggested.

*1) The new title of the manuscript is conflicting. “Using machine learning to reveal deeper insights into the drivers of in-hospital Acute coronary syndromes” indicates that the examined population did suffer from ACS during an hospitalization rather than being admitted for ACS. Please reframe to avoid confusion.*

- We fixed the title as suggested by the reviewer, and we welcome additional suggestions to improve the title of the manuscript.

*2) Line 72, 93 and 349: You refer to a risk stratification tool called MACE. To my knowledge MACE is a composite of major adverse cardiac events and not a risk stratification tool. In the reference that you added there is no risk stratification tool called “MACE” described. Please clarify what you are referring to.*

- We agree with the reviewer’s comment, therefore to avoid this confusion, we replaced it with other common risk stratification tools such EMMACE and GRACE with their relevant citations as examples.

*3) In the abstract, you claim that “anemia was the most important predictor of mortality,32 heart failure, and bleeding […]” however, in your results, you say that “in-hospital heart failure followed by initial hemoglobin values at admission and age, were the most important features for predicting the risk of mortality”. Please correct that in the abstract.*

- We fixed the sentence in the abstract as suggested by the reviewer.

*4) You explained the low rates of PCI in your ACS registry (0,35%) with accessibility and lack of necessity in most patients. Please report the rates of the subtypes of ACS in your baseline characteristics as it is very important to understand the quality of care the examined patients received in regard to the generalizability of your findings.*

*­*- It is very important to emphasize that the reported PCI in table 1 is an outcome PCI. In other words, this was the PCI rate for patients who during hospitalization suffered recurrent infarction as a complication during their hospital stay. Therefore, the rate of 0.35% is for the 14 patients whose denominator was 71 patients (who suffered re-infarction during their hospital stay). In other words, 14 of 71 (19.7%). That said, we added the ACS subtypes in table 1 as suggested by the reviewer.

*5) In table 1, the reference letter for the explanation of CVD in the figure legend is missing. Typically CVD also is the abbreviation for cardiovascular disease. If you really mean coronary vascular disease = coronary artery disease, the correct abbreviation would be CAD.*

*­*- We agree with the reviewer’s comment, here, we were using the expression CVD as an abbreviation for cardiovascular disease and therefore, we fixed it in the captions of table 1, as well as of figures 1, 3 and 4.
